# Assessment of the Quality and Mechanical Parameters of Castings Using Machine Learning Methods

**DOI:** 10.3390/ma15082884

**Published:** 2022-04-14

**Authors:** Krzysztof Jaśkowiec, Dorota Wilk-Kołodziejczyk, Śnieżyński Bartłomiej, Witor Reczek, Adam Bitka, Marcin Małysza, Maciej Doroszewski, Zenon Pirowski, Łukasz Boroń

**Affiliations:** 1 Center of Casting Technology, Łukasiewicz Research Network–Krakow Institute of Technology Contribution, Zakopiańska 73, 30-418 Krakow, Poland; dorota.wilk@kit.lukasiewicz.gov.pl (D.W.-K.); adam.bitka@kit.lukasiewicz.gov.pl (A.B.); marcin.malysza@kit.lukasiewicz.gov.pl (M.M.); zenon.pirowski@kit.lukasiewicz.gov.pl (Z.P.); lukasz.boron@kit.lukasiewicz.gov.pl (Ł.B.); 2 Faculty of Metals Engineering and Industrial Computer Science and Faculty of Computer Science, Electronics and Telecommunications, AGH University of Science and Technology, Al. Mickiewicza 30, 30-059 Krakow, Poland; bartlomiej.sniezynski@agh.edu.pl (Ś.B.); wikrecz@gmail.com (W.R.); doroszewski@gmail.com (M.D.)

**Keywords:** metal castings, mechanical parameters, application of machine learning methods

## Abstract

The aim of the work is to investigate the effectiveness of selected classification algorithms and their extensions in assessing microstructure of castings. Experiments were carried out in which the prepared algorithms and machine learning methods were tested in various conditions and configurations, as well as for various input data, which are photos of castings (photos of the microstructure) or information about the material (e.g., type, composition). As shown by the literature review, there are few scientific papers on this subject (i.e., in the use of machine learning to assess the quality of the microstructure and the obtained strength properties of cast iron). The effectiveness of machine learning algorithms in assessing the quality of castings will be tested using the most universal methods. Results obtained by classic machine learning methods and by neural networks will be compared with each other, taking into account aspects such as interpretability of results, ease of model implementation, algorithm simplicity, and learning time.

## 1. Introduction

The main assumption of this article was to conduct research on using machine learning methods to analyze microstructure photos and the values of mechanical parameters of castings in order to verify the correctness of their implementation. The analysis of the content of the publications found shows that this is an important research trend.

Tensile strength can be predicted on the basis of two different input data sources [1]:

- chemical composition of the metal and rolling process variables such as temperature and mileage;
- microstructure data.

A similar approach was used in [2], where a neural network was used and 20 variables such as chemical composition, heat treatment conditions, or test temperature were given at its input. In this way, 93% of the R-square coefficient values for yield strength (YS) and UTS were obtained. Another interesting approach was that presented in [3], where namely Bayesian neural networks were used again, but this time to predict the presence of microdamages in the casting before or during the casting process. With such data, it is possible to influence the casting process in such a way as to reduce the number of defects. In the BNN network, eight variables were used as input, such as geometric features (two variables), metallurgical quality (three vaiables), form quality (one variable), and two variables related to the process itself. Classification of microstructure is very difficult, and so far there have been not many scientific papers attempting to classify microstructures. Earlier articles usually separated the microstructure classification phase from the feature extraction phase [4]. With the advancement of deep learning (DL) methods, new opportunities have opened up. Many deep learning methods in various tasks achieve the best results that have been reached so far, so it is worth considering these methods. In the first study cited here, a new automated method was developed that uses Electron backscatter diffraction (EBSD) to efficiently identify and quantify ferrite micronutrients in complex microstructures of various steel grades [5]. The type of ferrite was identified for each grain, which was associated with the associated grain size. Different types of ferrites have different grain boundary confusion profiles–although this is angle-dependent–and this has been used in the study. On the other hand, in [6], a correlation approach based on EBSD and Light-optical microscopy (LOM) was used instead of using common methods independently. Using the grain orientation distribution in EBSD, the thresholds were manually established using a reference sample of the same microstructure plate. Similarly, in the case of LOM, the threshold was manually set and could be cross-validated. The second approach that was tested in [7] is to classify in terms of pixels by cutting out pieces of the original images with a sliding window method. We do this operation enough times to cover the entire input image. The output of such a network is a 3D matrix with the number of channels equal to the number of classes. Each pixel of this matrix has a value representing the confidence (or probability) of belonging to a given microstructure class. Then, the pixel classification step is performed by selecting the class with the highest probability (confidence, confidence) for each pixel. Next, all segments belonging to the original input image are merged together, and the maximum voting rule is applied to each object and assigned to it the class that most pixels have (i.e., the object is assigned the microstructure class with the maximum number of classified pixels inside the object). The work also uses class balancing and data augmentation. Balancing the classes was necessary because there were large discrepancies between the size of the examples in different classes, while the approach to this topic was original, namely, depending on the class size, different sizes of the step (stride) were used, so as to obtain a smaller or larger the number of examples cut. On the other hand, the data extension was conducted by rotating the images by 90°, 180° and 270°, thanks to which the set of images was expanded four times. Although the augmentation itself did not have a significant impact on the results, it achieved an increase of 2 percentage points. Thanks to this approach, it was possible to improve the best result so far by as many as 45 points. interest. Another work with promising results that also benefits from deep learning is [8]. The aim of the work is to segment structures in steel with a complex phase. The authors used the Vanilla U-Net and U-Net VGG16 networks in it, obtaining the best efficiency results, respectively 91.6% and 90.6%. These results were obtained for LOM image data. SEM image data were also used, but the efficacy was reduced by approximately 10 percentage points. Material properties such as strength, hardness, brittleness, or ductility are important in categorizing a material or component according to its quality, but these tests are conventionally expensive. Therefore, machine learning methods are considered helpful in predicting the properties of castings [9]. In the next work, supervised learning algorithms were used due to the characteristics of the data. The used support-vector ma-chine (SVM) tool is a supervised machine learning model that defines a hyperplane to separate examples belonging to two classes with a maximum margin [10].

## 2. Materials and Methods

**Figure 1:**
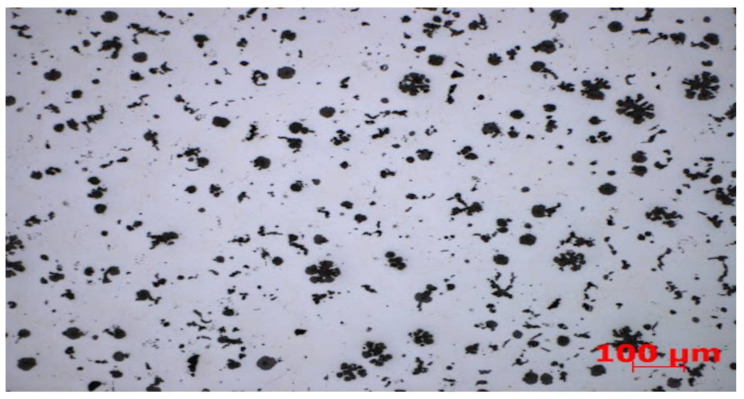
Sample photo of the microstructure. It shows an instance from the Rm set, where the photos are grouped by tensile strength. In this case, it is a low-resistance microstructure. Source: [11].

**Figure 2:**
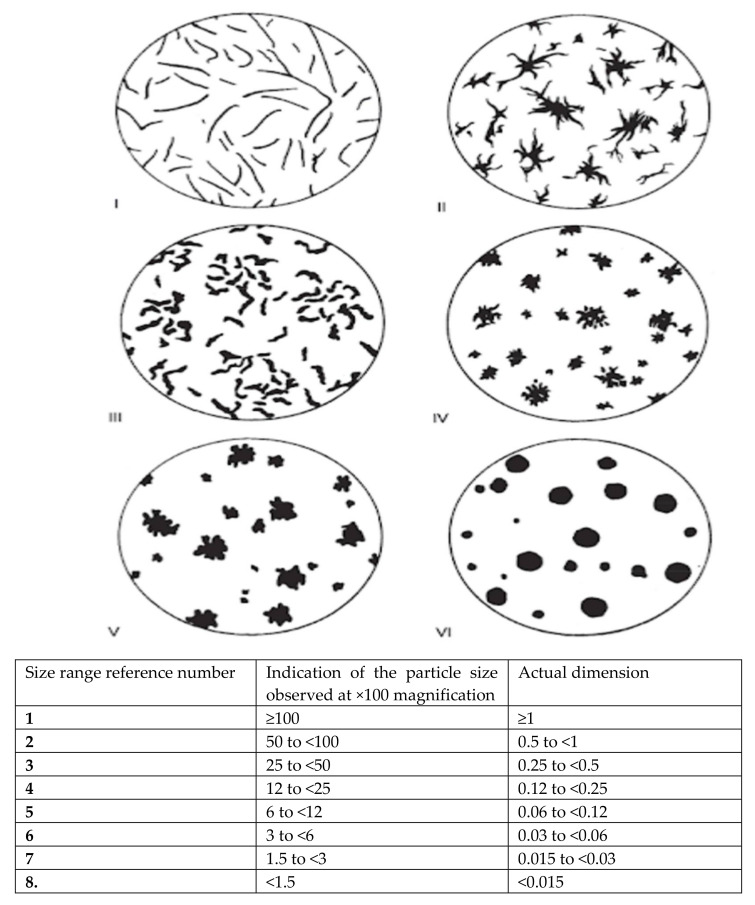
Reference images for the main graphite forms in cast iron. Source: [12]. Dimensions of graphite particle forms **I** to **VI**.

The collected data in the form of photos of the castings microstructure were tested for suitability. The images related to the data were divided according to the lower and higher value of the limit of elasticity. The subset containing the images with the lower limit of elasticity consisted of 68 instances, while the subset containing the images with the higher value of the limit of elasticity consisted of 47 instances. All images in this dataset were 1388 × 1040 in size. There were 115 instances in total in this collection. In another comparison, the data were divided according to the lower and higher value of tensile strength. The subset containing the images with the lower tensile strength consisted of 65 instances, while the image subset with the higher tensile strength consisted of 158 instances, hence 42 in total. There were 223 images in the examined set. This time, however, 138 instances are 1388 × 1040, while the remaining 85 instances are 2080 × 1540. All photos were taken with the LOM technique. Additionally, all photos are in RGB format, so they have three channels. After converting to grayscale, you can hardly see the difference. The vast majority of them have a hundredfold or five hundredfold approximation, while there are also photos with a twelve-and-a-half or fifty-fold approximation (although these are isolated cases). A close-up of a photo can be read from its file name, although photos with the same approximation may still differ slightly in their scale. Figure 1 shows an example of a microstructure. In the lower right corner of each shot there is a scale marked in red which shows the actual distance of the specified portion from the photo. Black structures on a gray background are a constant theme in these photos; these are the components of the structure of iron-carbon alloys, which we divide into six main categories based on their shape. These classes are shown in Figure 2. This split data will be used as input (i.e., the number of structures for each category) for traditional categorization models in research. This means that, like specialists in this field, we will try to predict the mechanical properties of castings based on the number of different forms in microstructures. Comparing the expertise with the knowledge extracted by the classifiers will be straightforward, making the models highly interpretable. Finally, you will be able to compare these results with those of the neural network.

Following the CRISP-DM methodology, the next step is data preparation. The process consists of the following sub-steps:

- data cleansing–including deleting photos that stand out too much from the rest (or that were noisy, or did not contain information that would enable their identification),
- transformations,
- data normalization, and reduction of photos to the same size (resolution) and the same scale.

Following the given order, at the very start, all photos of the lowest quality and those that were not made with the LOM technique or that represented other types of casts were removed.

**Figure 3:**
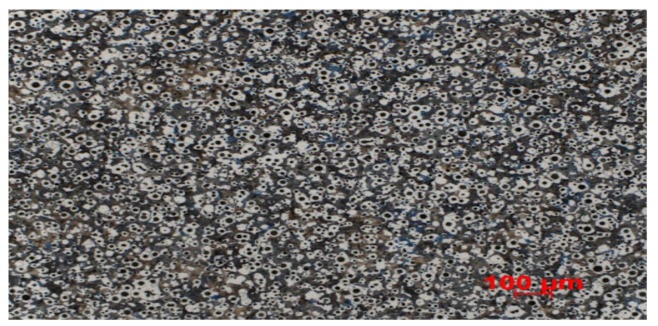
One of several rejected images. Comparing it with Figure 1, we can see that it would be very difficult to isolate the black structures. Source: [11].

An example of such an instance is shown in Figure 3. On the other hand, the second phase, i.e., data normalization, is a much more important and more difficult process. Work on this aspect has been divided into several stages:

1. normalization of approximation–as mentioned before, the photos had different approximations, so the first step was to bring them to the same approximation. It was decided that the best option is to zoom all photos to approximately 500×, which has several advantages including simplicity and an extension of the dataset,
2. and scale normalization–although the images were approximately similar, there were occasions when the scale length for one image differed from the scale length for another image.

## 3. Results

**Figure 4:**
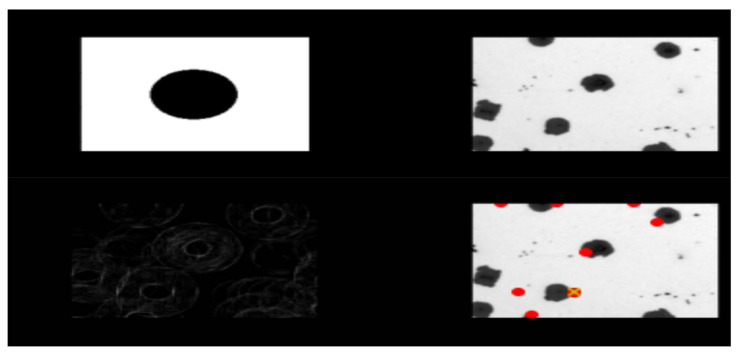
Detecting the shape shown in the reference image in the query image. In the lower right corner, the detected shapes are marked in red. The yellow cross shows the most relevant point. The reference image in this case is a black circle.

This research was divided into two major stages. The first was to identify single structures present in the images of microstructures. This involved delineating the contours of these objects, then cutting them out and creating a database from them, which required “manual” assignment of images to seven classes. Then, using this dataset, it would be possible to train a neural network that recognizes these shapes. Experiments began to test Hu moments and Haralick’s textures to classify entire microstructure images and find out which shapes are the most numerous. The next step was to test the generalized Hough transform. Following these tests, research into more sophisticated methods that could be applied more automatically began. The second stage consisted in assessing the quality of castings with the use of techniques developed in the first stage, using classic classifiers, but also using neural networks. First, Hu moments were used to extract features from the images that could be used to classify. The Haralick textures were tested analogously. These features were given as input to the classical classifiers. Then, comprehensive studies were carried out, which used the number of structures of specific classes found in the photos of microstructures, as well as classic classifiers. All obtained results were compared taking into account many aspects such as interpretability of results, ease of implementation, simplicity of algorithms, and learning time. In order to have an overview of the whole situation, the most effective neural network architectures used for the classification of images were also tested, and they were compared on a multi-plane basis with other results. Hu moments and Haralick textures were tested as a first approach, although they were also used to evaluate the quality of castings. The initial struggle showed that finding single structures of a given class with this method is possible, although not for all shapes. An example of a mistake is shown in Figure 4. GHT is believed to have advantages such as:

- resistance to partial or slight deformation of the form,
- resistance to the presence of other structures in the image,
- noise immunity,
- and the ability to find multiple occurrences of a given shape in the input image.

**Figure 5:**
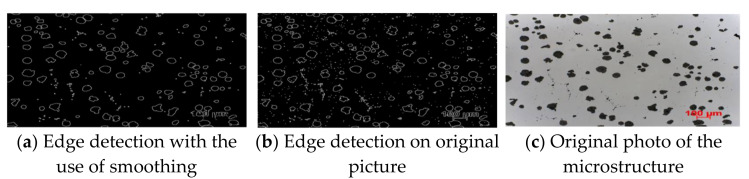
Edge detection with Canny filter.

Despite the fact that this method was created precisely to recognize mathematically defined forms (such as those present in our paintings), its operation did not meet our expectations. As shown in Figure 4, not all shapes were detected. The red dots representing the detected shape were sometimes shown on a gray background instead of on a black shape. However, these were not the only problem, and sometimes a given structure was marked multiple times. Therefore, it was decided to experiment with reference images, which could potentially improve the results. Nevertheless, this approach is not practical, because for different images, reference images should be selected in accordance with the scale of structures in the images. Additionally, even though there are only six classes of these structures, we would need many more examples to cover cases where the structure is distorted or overlaps with another structure. Thus, we already know that this method cannot be used in further research, and therefore it is necessary to continue in the search for an appropriate method. Subsequent techniques were more focused on cutting out individual structures and then categorizing each of them independently in the following phases. In the first step, an algorithm was developed to perform a simple structure count without distinguishing the shape class. The edges of the structures were detected using the Canny method [13]. Additionally, various blur techniques were tested, including averaging, Gaussian blur, median blurring, and bilateral filtering. Depending on the size of the kernel used, we obtained different blur effects. Figure 5 shows three photos, one of which is original (first from the right), and the other two which are the result of using edge detection.

**Figure 6:**
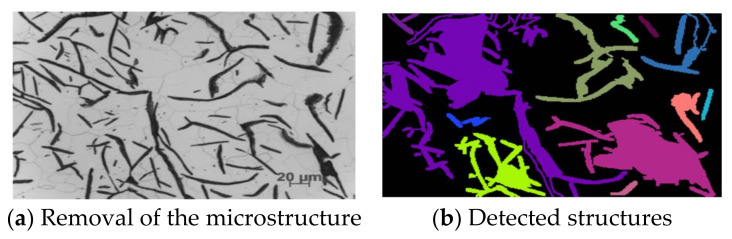
Canny Filter Operation. Each detected structure is marked with a different color.

The first image from the left uses anti-aliasing (5 × 5 kernel size), while the middle image does not. The detected structures were then counted; all the contours are shown in the first two pictures in Figure 5. The contours were counted in such a way that the area and perimeter were calculated for each object. Thus, contours with a small area or a small circumference can be discarded. All tiny, unimportant shapes are eliminated with this approach. During the trials, methods such as threshold, dilation, erosion, and morphology were also tested, however the best results were obtained when enumerating the structures in the original image. A strategy was adopted to cut out individual objects and place them on a white background. The structures were placed in the center of the 335 × 251 (W × H) white background image. Figure 6 shows how this process works.

The shapes that were treated as one object are marked with the same color. Structures in the image marked with the same color (and therefore considered as a single object) are cut out and placed against a white background. They will be used to train categorization models in subsequent phases. On the other hand, as you can see in the image above, the downside to this technique is that the structures are sometimes linked together and then treated as a single entity by the algorithm. This is most common with Grades I and II, which are stretched out in photos and are often in contact with each other. Attempts have been made to avoid this with algorithms such as erosion and thresholding, but these approaches have not achieved the desired results. In another example, the elements have been cut out separately. Only the combined shapes were cut out as one whole. Unfortunately, there is no universal solution for this; once a problem is solved, a new one appears, for instance less visible structures not being cut out, or the detection of faded structures that should be omitted. Objects cut in this way are introduced to the input of the neural network, which will be trained to categorize these structures. We have seven different classes in our collection. Six of them are the shapes highlighted in the standard [14], while the author added a seventh grade to distinguish these structures from objects that were poorly cut or containing the scale that can be found in all microstructure photos. All cut elements were stored locally and arbitrarily assigned to classes when the dataset was created. At this stage of the research, we already have personally prepared data on individual structures. Again, experiments could be commenced with the simplest methods such as Hu moments, Zernike moments, and Haralick textures, along with classical classifiers, although this approach has already been tested in [15] and it has been shown that in the case of such sets of algorithms and available data, the results are comparable to those we obtain when using classic classifiers with input in the form of pixels. Therefore, here we will go straight to the more advanced methods. For this purpose, it was decided to use neural networks. In particular, transfer learning was applied using the VGG19 network. The implementation of this architecture was obtained from the Keras library. During the training, this network was excluded from training. In addition, its tip was not included, but several additional layers were added, which allowed us to train the network for our data. The Adam optimizer was used, the batch size was 1, while the number of epochs was 100. Such a network on average achieves an efficiency of about 82% for test data (in the case of training data, the accuracy ranges from 86% to 95% efficiency, depending on the configuration), the value of the metric F1 is 0.79, and logistic loss is 0.66. These are better results than in the previously quoted work [15] due to the fact that we are working on corrected data, which was described in detail in the quoted work. After conversion to the relative error, we get errors of 10%, 52%, 26%, 50%, 49%, and 1% for classes I to VI, respectively (no class “0” in the test data due to their low frequency). However, errors are mainly due to the similarity of these forms within “adjacent” classes such as classes V and VI (in the figure these are classes 4 and 5), where almost half of the shapes in class V are classified as class VI. The similarity of classes is more evident in the case of real data, as most of these shapes contain features of many classes (they are difficult to attribute to one specific feature). The discrepancy in prediction accuracy between classes is most likely related to an imbalance in the amount of data available for each class. On the other hand, the results appear to be much better when using training data for evaluation. After calculating the relative errors, we achieve the following results: 36% (class “0”), 0.4%, 20%, 7%, 19%, 38%, and 3%. We can observe that the training data have far fewer relative errors, indicating that the results can still be improved. Data balancing may be a good direction, because, as you can see, the best results are obtained for classes whose number of instances was the largest in the set (except for class V, although examples of this class are confusingly similar to examples from class VI). Thus, a strategy was adopted to extend the data from these classes, which at the same time have the lowest effectiveness and the fewest examples. Hence, the following classes will be extended: “0”, II, IV, and V. The reflection method will be used, which does not change the size of the image. The horizontal reflection method will also be used this time. In addition, several other approaches were tested, such as superimposing noise on extended examples of data, as well as removing gray from the images. The manipulation of the data did not positively influence the classification result architecture, for which the best results were achieved. Given the imbalance of the classes and the fact that these data were prepared by hand by the author of this work (who is not a metallurgical specialist), in order to improve the results of this classification it is likely that an expert should prepare it in the field. In further research, the architecture will be used together with the data for which the highest results were obtained, i.e., the VGG19 neural network with the originally prepared images of the structures. In testing, there are sample photos that represent the whole process well; with these, it looks like operations are performed for each input photo and then classified by the architecture presented in this chapter. Therefore, errors may occur, such as failure to detect a given structure or misclassification of the structure. The network classifying shapes together with the whole sequence of recognizing individual structures brings quite good results. Visually, there is nothing to criticize here. However, it is often the case, as already mentioned, that these structures are very similar to each other and their preparation by a specialist in the field of metallurgy could bring about a significant increase in the efficiency of the model. The last attempt to improve the performance of the model was to eliminate the smallest structures from the classification. They are the most problematic because visually they are practically the same. Additionally, the author himself had a problem with assigning these structures to a specific class. After removing these structures, we obtained an efficiency of 82%, while the value of the logistic loss was 0.8, which is a very good result. Class II and V recorded the highest losses, i.e., 7 and 10 percentage points, respectively. On the other hand, classes IV and VI recorded the highest profits, as much as 33 and 6 percentage points, respectively. After researching the topic presented in this chapter, many interesting conclusions can be drawn:

GHT achieved quite precise results, but it had many restrictions: first of all, the necessity to use an identical reference image to the one searched for, although GHT method can be effectively used for other applications, as when Gaussian blur was used, cracked structures were detected better (because cracks were not were considered structures). Furthermore, classes V–VI were gaining in effectiveness, while other classes (mainly I–II) were losing.

**Figure 7:**
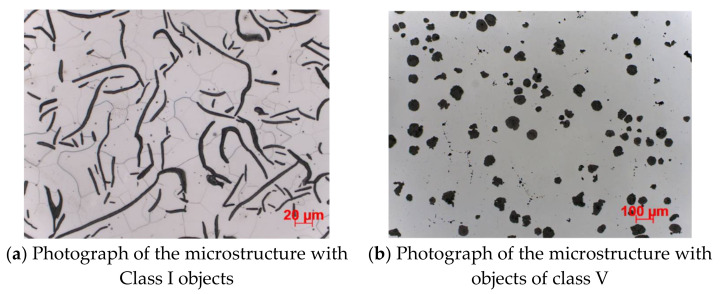
Two sample photos of microstructures. Their textures and shapes in this location are radically different. It’s important for used artificial intelligence methods.

The thresholding was also tested, which consists in binarizing photos, i.e., assigning them black or white pixels depending on the previous value of these pixels. This was to prevent cracks from being treated as a structure, however, overall effectiveness also decreased. This is the second broadest area of research, as it is the main point of this research and the topic of this work. All tests related to casting quality assessment are collected and presented here. The experiments were presented in the order in which they were performed, i.e., from the simplest methods to the most complex ones, which at the same time returned the best results. The study began by checking the effectiveness of classic classifiers on data in the form of Hu moments and Haralick textures. Then, classic classifiers were tested, which received previously prepared data as input in the form of the number of structures of individual classes. Lastly, the effectiveness of neural networks was examined, as well as an additional hybrid approach, in which the result of the VGG19 network classification was added to the input of classic classifiers (thus, they received the number of structures and the result of the VGG19 network classification). The first approach was to use Haralick’s textures and Hu moments to identify the structures in the image. The implementation of the Hu moments method is taken from the opencv-python package, while the Haralick texture implementation is taken from the mahotas module. It may seem that the use of these algorithms is correct and will achieve the results you want because they were designed specifically for this purpose. Figure 7 shows two images of different microstructures that could potentially be identified using the described techniques.

The first experiment used Haralick textures, Hu moments, and support vector machine (SVM) and random forest (RF, RFC) models. The tests were carried out on a set of data classified according to tensile strength. Unfortunately, such a configuration of models and methods did not bring the expected results. The tests were performed using a cross-validation, namely a k-fold test, in which the original sample is divided into k subsets, and each of these subsets is used as the test set, while all others are used as the training set. Then these k results are averaged. The results are presented in Table 1. As we can see, the results for most of these methods are around 71%. A slightly worse result for random forest may be due to the application balancing the class weights, however, the dataset consists of 3358 high-tensile photos and 1337 low-tensile photos. Therefore, the number of high-strength photos is exactly 71% of the total data, so the classifiers, instead of actually recognizing the data, can actually match the frequency of occurrence of a certain class of photos. Hence, additional tests were performed using augmentation.

Specifically, the reflection method was used on a smaller class (and therefore low strength photos), so that the ratio of the number of high strength photos to the number of all photos dropped from 71% to 55.7%. Additionally, in order not to artificially increase the accuracy due to the similarity of the artificially generated and original data, it was decided to impose noise on the synthetically generated data. Comparing the results obtained in these three experiments, one can certainly come to several conclusions. First, it is clear that the results between the tree models and the vector machines differ significantly. In the first experiment, the results for the SVM are better from 1 to even 12 percentage points. In the other two experiments, the results for the SVM are much worse than those for the random forest, in an extreme case by as much as 30 percentage points. Second, it can be seen that in most cases the results for Hu moments are the worst, which can be explained by the fact that it is the simplest method. In contrast, we also see the impact of data augmentation as well as data noise. It can be assumed that the most reliable results were obtained in the third experiment, because first of all, the effectiveness of the algorithm does not coincide with the ratio of the class size, and the results do not differ inexplicably. After all, it is hard to fully assess what really influenced these results. Additionally, this approach has a very low interpretability, i.e., it is hard to judge why the model chose one decision over another. On the other hand, the advantage of this approach is the execution time (model learning time). Despite setting the SVM parameters for many iterations, as well as setting a relatively large number of trees for the random forest algorithm, the training time of these models is in the order of several seconds. As we can see, even the simplest methods give some positive results, so it was decided to use a slightly more complex strategy, which may increase the effectiveness. In the next step, the effectiveness of classic classifiers in the assessment of the quality of castings (i.e., prediction of the tensile strength of castings) was tested. As input data, the number of structures of individual classes was used, which are obtained in the process. Hence, a table of seven numbers is fed to the input of classic classifiers, each of which corresponds to the size of the appropriate class (i.e., the number “zero” corresponds to the number of objects of class “0”, etc.), and based on these data, they try to predict the tensile strength of the castings presented in the photos to which the data relates. Approaches were also tested in which, instead of absolute numbers, the percentage of structures of individual classes was entered as input, as well as strategies in which the input was the result of the softmax function on the absolute frequencies of the structures of individual classes. For this purpose, the most popular classifiers were used, such as support vector machine (SVM), decision tree (DT), random forest (RFC), logistic regression (logit), multi-layer perceptron (MLP), and AdaBoost. The first approach will be discussed, in which the SVM, i.e., the support vector machine, was used as a classical classifier. First, a strategy was tested in which the absolute number of structures of each class was given as input to the SVM. The studies were carried out using cross-validation and mesh searching. As a result, a model was obtained that achieved 83.3% accuracy for the test data and about four percentage points more for the training data (86.7%). The value of the logistic loss was 0.39. This means that the model has not been overtrained or under-trained to the data, which is also prevented by cross-validation. In any case, the results appeared to be high, especially as they were based on a model that was less successful (by half a percentage point). Another idea tested was a strategy in which, instead of absolute numbers describing the multiplicity of the structures of the respective classes, the percentage share of the structures of each class was given at the input. This time, the effectiveness was 78.7%, which is exactly four percentage points less than in the case of the number of structures. The mean precision value was 0.8, which is also worse than in the previous case. Furthermore, the value of the logistic loss was greater, and amounts to 0.47. Hence, the graph is not even required, because after the numerical results alone, we can definitely say that the previous approach worked better here. Trying to explain such discrepancies, it is really hard to come to a meaningful explanation of this situation. It might seem that the results should be much more similar, due to the nature of the input data. The percentage share of individual structures is nothing more than normalized results taking into account the number of structures of a given class in relation to all others, and yet, the results are much worse. However, there was one slight difference; namely, there were nine photos for which not a single structure was detected, hence it was impossible to calculate the relative share of the structures of each class, and therefore it was necessary to eliminate them from the collection. Either way, these are only nine examples out of all 4695, hence this fact can be ignored. The last strategy tested was the strategy in which the input of the classic classifiers is the result of the softmax function on the original data (i.e., the multiplicity of the structures of individual classes). In this case, we reached an accuracy of 79%, an average precision value of 0.72, and a logistic loss value of 0.51. As we can see, out of all three strategies used here, the absolute number of structures of individual classes works best, so in further research we will mainly focus on this type of data. However, the data are highly unbalanced. As a reminder, there are 3358 photos representing castings with high tensile strength, while there are 1337 of those with low tensile strength. In terms of terminology, it is only a “slightly unbalanced set”, although it is worth checking whether this imbalance affects the results. The technique of balancing the sets, namely by eliminating redundant examples over the class represented from the data, resulted in 1337 examples of both high and low strength microstructures. However, after balancing the classes, the problem persisted. The same tests and values were carried out; all the metrics worsened, although the table of errors after this treatment looked a little better. Hence, it is known that the shift towards the high-strength class is not a matter of class imbalance. In the results, we can see that the best results are obtained for the original input data, i.e., in the form of the multiplicity of the structures of individual classes. Hence, in subsequent studies with the use of other classifiers, we will mainly focus on this type of data. Additionally, optimization was performed in an attempt to obtain the best possible result. Hyperparameters involved a slightly different search than in the case of the previous module. This strategy produced the best results when applied with cross-validation. For the data in the form of the number of structures, we achieve 84% accuracy, 0.88 average precision value, and 5.66 logistic loss value. For the CART algorithm, as input data, the absolute multiplicity of the structures of individual classes was used. The tests were divided according to the depth of the tested trees. The input dimension is seven. First, research was carried out for trees with a depth equal to one. For such trees, the average accuracy of the model is 78%, which is already a very good result. The most important here are type III structures. Considering only this one type of structure, we achieve the accuracy of the mentioned level of 78%. Now, if we look at the results for a tree with a depth equal to two, the accuracy is 81.2%. As we can see, class III is again the most important class, but we also have classes I and VI. A logical explanation for this may be the fact that the structures of these three classes differ most visually from the others. For a tree with a depth of three, we achieve the accuracy of 81.3%, which is only one-tenth of a percentage point more than for a tree with a depth of two. This may indicate that the model is too tailored to the data, or that it is poorly generalized. For greater tree depths, no increase in accuracy was noted. Meanwhile, we will move on to the next decision tree generation algorithm. In addition to the CART algorithm, the ID3 algorithm was also tested, the implementation of which was taken from the decision-tree-id3 library. Such features as learning time, time to return results, or accuracy were confronted with each other. Let us consider the model learning times depending on the algorithm used and the tree depth. The training time is significantly longer for the ID3 algorithm, and it increases with increasing tree depth. This is a known property of these algorithms, as in general the CART algorithm works faster, especially when we use an optimized version of the CART algorithm. The next metric is the time it takes to achieve the result. The CART algorithm looks better in this comparison as well; its output is returned almost immediately, while the results of the tree built using the ID3 algorithm take longer to wait, and this time is even longer when dealing with deeper trees. The last metric we will use to compare the two algorithms will be accuracy, and we will try to investigate which algorithm generates more accurate trees in terms of classification.

**Figure 8:**
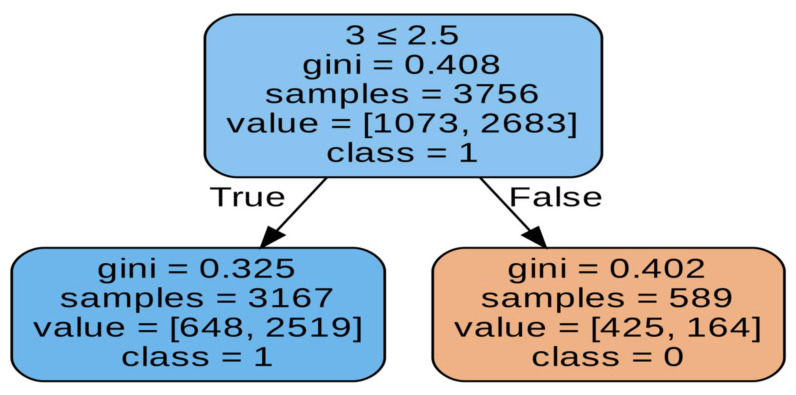
Visualization of the decision tree.

**Figure 9:**
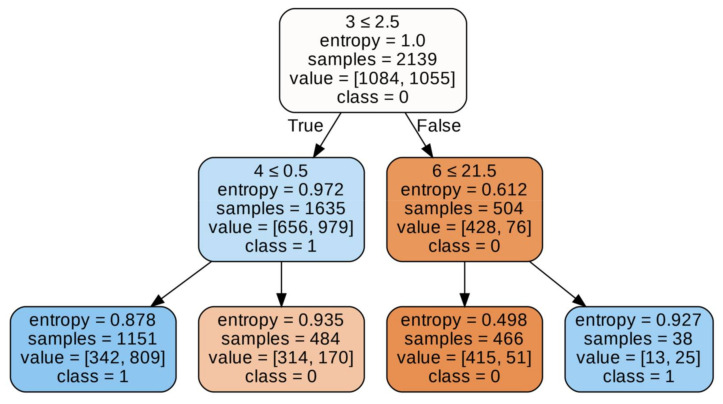
Presents a visualization of a tree constructed with the CART algorithm using hyperparameter optimization.

As you know, different tree generation algorithms use different tree splitting criteria. Thus, the ID3 algorithm uses entropy or information gain. On the other hand, the CART algorithm can use Giniimpurity, for example. Hence, there may be discrepancies in the accuracy of these trees. It cannot be clearly stated which of these approaches is more accurate, because depending on the depth, the model achieves slightly better results. However, taking into account the previous factors, it seems that the CART algorithm may actually be a better choice, mainly if we are training deep tree and are on time, including the time to obtain results. Finally, before we present a summary table of all tests related to the decision tree, we will look at the results obtained with a decision tree constructed using the CART algorithm, after we have balanced the data. The CART algorithm using hyperparameter optimization of the accuracy of this model is 73.5%, so we recorded a decrease of 7.7 percentage points. We can see that the abundance of the Type III structure is decisive here on Figure 8. The condition is shown in the first line of the first decision block. We check whether the type III structure is less than or equal. On this basis, the casting strength is determined. On Figure 9 accuracy of model is 73.5%, so we recorded a decrease of 7.7 percentage points.

When there are a number of these structures (more than two), we can say with great certainty that the structure has a low tensile strength. Otherwise (i.e., when there are less than three structures), the classification is not that exact. Hence, it can be concluded that the presence of type III structures causes the structure to have a low tensile strength. The classification is slightly shifted towards high strength (class 1). This can be explained using the tree. When there are less than three type III structures, the accuracy of our classification is about 69% (left sub-tree). And since most of the structures present there are of high strength, this explains the shift towards this class. However, we also see a decrease in the effectiveness of detecting high strength, which was due to a reduction in the amount of data on this property. Although the results are now more reliable, we can see that when using the multiplicity of structures alone, we are not able to state with a very high efficiency what the strength of this structure is. Hence, the thesis that neural networks assessing this feature of castings on the basis of pixels may achieve higher efficiencies seems even more probable; it contains the results of most noteworthy tests, including those not described in detail here. Of course, we achieve the best results with deeper trees. However, two things are worth noting above all else. First, after balancing the data, a significant drop in effectiveness was noted, which has already been discussed above in this section. Secondly, the highest accuracy is obtained for the data in the form of the absolute number of structures of individual classes, slightly worse results when we give the percentage share of structures of individual classes as input, and the worst results are obtained when we use the softmax function. In a way, it is understandable that we obtain slightly worse results for the softmax function, as it removes some information from the data. On the other hand, the decrease for the data in the form of percentage share is unusual, although similar results were obtained for the SVM model. A simple explanation may be based on the fact that the strength of structures is actually more influenced by whether the structures of a given class occurred at all, rather than the ratio of their numbers to other classes. Another classic classifier which has been tested in the assessment of the tensile strength of castings is a random forest. The results obtained with the use of hyperparameter optimization and cross-validation on original and balanced data will be presented here in detail. On the other hand, the table at the end of the subsection will present all the significant results, the tests of which were analogous to those presented. First, the model for the original data was tested. This model obtained the efficiency at the level of 83%, the average precision value at the level of 0.91, and the value of the logistic loss function at the level of 0.4. The value of the F1 metric was 0.89. Generally, it can be stated that the results are as correct as possible. Namely, it is a shift towards a high-endurance class. Therefore, these tests were repeated for balanced data and compared with the previous results. When creating balanced data, all low-strength examples are included in the set (because there are definitely fewer of them), while as many examples of high-strength examples are randomly selected, in order to match the number of the previous class. The results that were achieved for this comparison are an accuracy of 80.9%, an average precision value of 0.85, a logistic loss function value of 0.47, and a F1 metric value of 0.81. As we can see, there has been a decrease in these metrics, however, it is not as large as in the case of decision trees. Balancing the data brought the expected effect. In addition, it showed that while there were actually drops in effectiveness, they were very small, which proves the effectiveness of this approach. Finally, we present a table that summarizes most of the studies that have been conducted that have achieved significant results. As we can see, the trend in effectiveness for the different types of input is maintained. Again, the best results are obtained with the data in the form of the number of structures, although the results between the different types of input do not differ significantly. In addition, the results for balanced data are only 2.1 percentage points inferior to the best results obtained with the random forest. The next model that has been tested is logistic regression. As it turned out, somewhat unexpectedly, the results for the logistic regression were quite decent. Starting with the unbalanced data, the following results were obtained: 83.1% accuracy, 0.91 of the mean value of the precision, 0.41 values of the logistic loss function, and 0.88 values of the F1 metric. For the balanced data, we obtained the following results:

- 82.6% accuracy,
- 0.85 average precision value,
- 0.52 logistic loss function value,
- and F1-0.8 metric value.

The table of errors shows the balance of the data causes the values of the metrics to drop slightly, but this time the drops are smaller than in the case of the previous classifiers. Comparing the results obtained with the use of logistic regression and those by the random forest, we can see that both the best results were achieved by logistic regression (by 0.1 percentage point), and the balanced data here exceeded the random forest by as much as 1.7 percentage points.

Another model tested was the multilayer perceptron. It is a slightly different model from all those presented in this chapter, as it is the most popular type of artificial neural network. The results for unbalanced data are as follows:

- accuracy: 82.6%;
- average precision value: 0.85;
- value of the logistic loss function: 0.41;
- value of the metric F1: 0.88.

In addition to accuracy, the values of all other metrics are the best when compared to the other models, with respect to balanced data. Additionally, the results are more even for the various input types compared to the previous models. 

The last model tested in this statement is the AdaBoost algorithm. It consists of many so-called weak classifiers, and with each iteration, the weight of poorly classified observations is increased. In the scikit-learn library, the default weak classifier is the decision tree, and there are 50 of them. One would expect high performance of this model, not much worse than that of the random forest. The results for balanced data are as follows:

- accuracy: 83.4%;
- average precision value: 0.91;
- value of the logistic loss function: 0.41;
- value of the metric F1: 0.88.

**Figure 10:**
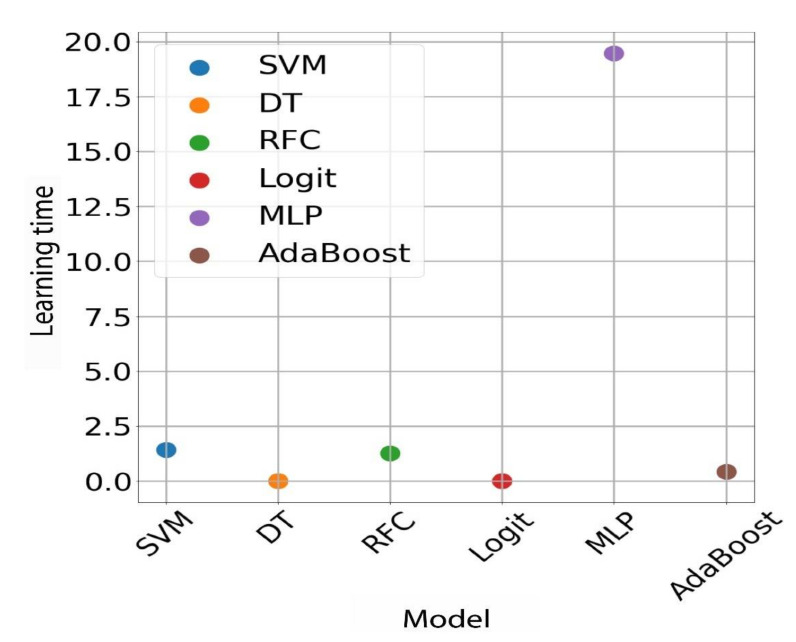
Average times of returning the results for individual models. Calculation times for unbalanced data.

This is the highest accuracy that has been achieved for this type of data. The table below shows the results that were achieved with this model for the different input types. There was a significant drop in effectiveness; a possible explanation is that the model is overfitting with the training data. As you know, reinforcement-based models tend to do so, all the more so as the advanced data contains 40% of all available data. As can be seen, for unbalanced data, support vector machine, random forest, and AdaBoost machine models perform well, which is the expected result as these are very popular algorithms, generally considered robust. In contrast, for balanced data, undoubtedly the best model is the multilayer perceptron, which achieved the best values for all metrics. In addition to this extensive comparison of models based on performance metrics, graphs were also prepared showing the average training times and the times of returning the results for individual models. As we can see, it takes by far the longest to train a multilayer perceptron model, which is understandable because it is actually a neural network with many neurons per layer and hundreds of iterations. Then, we have models of the support vector machine and the random forest. SVM is based on the kernel function, and big data can run slowly. On the other hand, the random forest consists of hundreds of individual models (so-called weak classifiers); in this case they are the so-called decision stumps, i.e., decision trees with a depth equal to one. Training the RFC model is more than 400 times longer than a single decision tree, which would be consistent with the fact that the tests used a random forest model with 473 trees. Next, we have the AdaBoost model. This model also consists of many weak classifiers, and they are also decision stumps. Since there are 50 of them, its learning time is correspondingly longer. The other two models, i.e., the decision tree and logistic regression, have by far the shortest learning times (close to zero) (Figure 10). 

These are the simplest models that do not require many calculations. In this case, the research confirmed what we knew from the theory. Below is a chart of the times taken to obtain results for the same models. For this chart, the results are also in line with expectations. There is a low result return time for a neural network, although neural networks are very fast if we want to obtain a prediction for a single input instance; this only takes a few multiplication operations, and we obtain the result. However, the longest wait time was for the result of a random forest. This is understandable, as it consists of more than 450 decision trees. Then, we have SVM and AdaBoost models. The explanation for AdaBoost is analogous to the case of the random forest. However, again the shortest time should be for the results of a single decision tree and logistic regression. As you know, these algorithms are very simple mathematically. Depending on the requirements, the user should independently choose which algorithm will be best used. In our case, where the data were not too much and not very complicated, all the classifiers actually met their requirements. However, as research has shown, these models differ from each other, and for large data, these time differences can become very large and be of great importance for the entire process (learning and evaluation).

## 4. Summary and Conclusions

During the course of this project, there were many problems that needed to be resolved. Leaving aside the literature review, the next step was to prepare the data appropriately. First of all, it was necessary to reduce the photos to the same scale and zoom in, as well as remove unnecessary elements from them (such as the scale size). In addition, further data were artificially generated using augmentation techniques that could be useful in the future. After handling the work related to the data, it was possible to move on to more serious issues that were the subject of this work. Chronologically, the first problem was the classification of structures. The first approach tested was the generalized Hough transform, which is a method used to find the shapes shown in the reference image in the query image. Admittedly, after a few modifications of the procedure, the visual results looked great, but it was a very impractical approach, as for each image of the microstructure we would need at least six reference images, and potentially even more, because in fact the structures in the photos were slightly different from those indicated in the standard. Although this method was close to the solution, it was only the next tested methods that met our expectations, namely algorithms from the OpenCV library, such as smoothing, thresholding, and most of all the Canny edge detection method [13]. Thanks to these methods, it became possible to cut virtually all structures and then manually classify them in order to create a new database. The best result that was achieved is 82.2% accuracy. The results look great visually, although verifying them with a table of errors, it turns out that there are slight misconceptions. First of all, the results for five of the seven classes look decent, while for the other two classes (II and IV) they are slightly worse. Many tests have been carried out, and unfortunately everything indicates that it is not possible to achieve much better results with this data. Then, the research that was the main topic of this paper is presented. In order to classify the binary tensile strength of the castings, the methods presented in the previous chapters were used. However, the first method tested was the Hu moments and the Haralick textures, which seem to be the ideal tool for this purpose, as long as the structures present in the photos have an effect on strength. Additionally, classical classification models were used. Unfortunately, although the results were varied, from low (55% accuracy) to high (70% accuracy), the results are still not fully satisfactory. Then, the classic classifiers were tested using the data generated by the authors and the classic classifiers. This is the most extensive research point. As it turned out, this is a very effective method, as it has managed to achieve an efficiency of as much as 83.4% accuracy. In addition, these methods are highly interpretable, and therefore could serve as an aid to specialists who would like to use photos to assess the strength of a given material. In recent tests, the effectiveness of currently very popular neural networks (in particular VGG19), as well as the connection of neural networks with classic classifiers, were tested. As it turned out, however, it was not possible to achieve better results; they were just on par with the best results obtained so far. However, we know that neural networks work much better for large amounts of data, which was a minor problem here (described later). As further work on this topic, the need to better prepare the learning data, for example with the help of specialists in this field, can be considered. For all of these methods, it is most important that in a given class there are only the correct examples, as possible. On the other hand, the problem of the lower rank (albeit also important in order to obtain the best possible results) is the balance of the classes. In addition, the author chose only a fraction of the cut structures (around 1500), because after bringing the photos to the same scale (i.e., photos with an approximation of 100× were reduced to the approximation of 500×, thus obtaining another 25 from such a single photo) there are as many as 4797, of which on average 20 structures are cut from each photo. Therefore, the number of possible structures obtained from this set may be even greater than 100,000 (say: one hundred thousand), so approximately 1.5% of the available data was used. Hence, it can be seen that there is still a lot of room for improvement in terms of data. However, we can see that both classical models and neural networks achieved decent effectiveness. In particular, at the moment, it seems justified to use classic models (which in fact also indirectly use the effects of the VGG19 network), which could be used by experts in this field as an aid in justifying the decision on the strength of a given material. In the case of a neural network, here it seems necessary to collect more data and repeat similar tests to show that they can achieve even better results. The article focuses on selected aspects. It should be remembered that the results are a contribution of the system to cast iron production.

## Figures and Tables

**Table 1:** Results of binary classification with the use of classical classifiers, using Hu moments and Haralick textures.

Model	Input Type	Class Weights	Accuracy
SVM	Hu ^a^	-	71.5%
SVM	Haralick ^b^	-	71.5%
SVM	Hu+ Haralick ^c^	-	71.5%
RFC	Hu	sustainable	58%
RFC	Haralick	sustainable	70%
RFC	Hu + Haralick	sustainable	70.1%

## References

[1] Yang Y.Y., Mahfouf M., Linkens D.A., Zhang Q. (2016). Tensile Strength Prediction for Hot Rolled Steels by Bayesian Neural Network Model. IFAC Proceedings Volumes.

[2] Wang Y., Wu X., Li X., Xie Z., Liu R., Liu W., Zhang Y., Liu Y.X.C. (2020). Prediction and Analysis of Tensile Properties of Austenitic Stainless Steel Using Artificial Neural Network. Metals.

[3] Penya Y.K., Bringas P.G., Zabala A. Advanced fault prediction in high-precision foundry production. Proceedings of the 2008 6th IEEE International Conference on Industrial Informatics.

[4] Azimi S.M., Britz D., Engstler M., Fritz M., Mücklich F. (2018). Advanced Steel Microstructural Classification by Deep Learning Methods. Sci. Rep..

[5] Shrestha S.L., Breenb A.J., Trimby P., Simon G.P., Ringerab J., Cairneyab P. (2013). An Automated Method of Quantifying Ferrite Microstructures Using Electron Backscatter Diffraction (EBSD) Data. Ultramicroscopy.

[6] Schneider A., Britz D., Webel J., Mücklich F. (2017). Identifying and Quantifying Microstructures in Low-alloyed Steels: A Correlative Approach. Metall. Ital..

[7] Britz D., Pauly J., Mücklich F. (2016). Advanced Microstructure Classification Using Data Mining Methods. Comput. Mater. Sci..

[8] Durmaz A.R., Müller M., Lei B., Thomas A., Britz D., Holm E.A., Eberl Ch Mücklich F., Gumbsch P. (2021). A Deep Learning Approach for Complex Microstructure Inference. Res. Sq..

[9] Stoll A., Benner P. (2021). Machine Learning for Material Characterization with an Application for Predicting Mechanical Properties.

[10] Burns A., Dobbing B., Vardanega I.T. (2014). Machine Learning and AI via Brain Simulations.

[11] Pirowski Z. (2017). Opracowanie Innowacyjnych Elementów Roboczych Maszyn Sektora Leśnego i Przetwórstwa Biomasy w Oparciu o Wysokoenergetyczne Technologie Powierzchniowej Modyfikacji Warstwy Wierzchniej Elementów Odlewanych.

[12] (2018). Mikrostruktura Żeliwa –Część: Klasyfikacja Wydzieleń Grafitu na Podstawie Analizy Wizualnej.

[13] Canny J. (1986). A Computational Approach to Edge Detection. IEEE Trans. Pattern Anal. Mach. Intell..

[14] Bickel S. ECML-PKDD Discovery Challenge 2006 Overview. Proceedings of the ECML-PKDD Discovery Challenge Workshop.

[15] Reczek W. (2021). Raport z Pracowni Problemowej: Analiza Obrazów w Celu Identyfikacji Parametrów Materiałów Odlewniczych.

